# Federated Learning on Clinical Benchmark Data: Performance Assessment

**DOI:** 10.2196/20891

**Published:** 2020-10-26

**Authors:** Geun Hyeong Lee, Soo-Yong Shin

**Affiliations:** 1 Department of Digital Health Samsung Advanced Institute for Health Sciences & Technology Sungkyunkwan University Seoul Republic of Korea; 2 Big Data Research Center Samsung Medical Center Seoul Republic of Korea; 3 Department of Intelligent Precision Healthcare Convergence Sungkyunkwan University Suwon Republic of Korea

**Keywords:** federated learning, medical data, privacy protection, machine learning, deep learning

## Abstract

**Background:**

Federated learning (FL) is a newly proposed machine-learning method that uses a decentralized dataset. Since data transfer is not necessary for the learning process in FL, there is a significant advantage in protecting personal privacy. Therefore, many studies are being actively conducted in the applications of FL for diverse areas.

**Objective:**

The aim of this study was to evaluate the reliability and performance of FL using three benchmark datasets, including a clinical benchmark dataset.

**Methods:**

To evaluate FL in a realistic setting, we implemented FL using a client-server architecture with Python. The implemented client-server version of the FL software was deployed to Amazon Web Services. Modified National Institute of Standards and Technology (MNIST), Medical Information Mart for Intensive Care-III (MIMIC-III), and electrocardiogram (ECG) datasets were used to evaluate the performance of FL. To test FL in a realistic setting, the MNIST dataset was split into 10 different clients, with one digit for each client. In addition, we conducted four different experiments according to basic, imbalanced, skewed, and a combination of imbalanced and skewed data distributions. We also compared the performance of FL to that of the state-of-the-art method with respect to in-hospital mortality using the MIMIC-III dataset. Likewise, we conducted experiments comparing basic and imbalanced data distributions using MIMIC-III and ECG data.

**Results:**

FL on the basic MNIST dataset with 10 clients achieved an area under the receiver operating characteristic curve (AUROC) of 0.997 and an F1-score of 0.946. The experiment with the imbalanced MNIST dataset achieved an AUROC of 0.995 and an F1-score of 0.921. The experiment with the skewed MNIST dataset achieved an AUROC of 0.992 and an F1-score of 0.905. Finally, the combined imbalanced and skewed experiment achieved an AUROC of 0.990 and an F1-score of 0.891. The basic FL on in-hospital mortality using MIMIC-III data achieved an AUROC of 0.850 and an F1-score of 0.944, while the experiment with the imbalanced MIMIC-III dataset achieved an AUROC of 0.850 and an F1-score of 0.943. For ECG classification, the basic FL achieved an AUROC of 0.938 and an F1-score of 0.807, and the imbalanced ECG dataset achieved an AUROC of 0.943 and an F1-score of 0.807.

**Conclusions:**

FL demonstrated comparative performance on different benchmark datasets. In addition, FL demonstrated reliable performance in cases where the distribution was imbalanced, skewed, and extreme, reflecting the real-life scenario in which data distributions from various hospitals are different. FL can achieve high performance while maintaining privacy protection because there is no requirement to centralize the data.

## Introduction

### Background

Traditional machine learning and deep learning require a centralized dataset to train a model. Therefore, such methods not only require data transfer to collect data from many devices, people, or institutions but also have a high computational cost because they must be trained on large datasets. When collecting privacy-sensitive data such as medical data, privacy protection is a major hurdle. Centralized databases are the main targets of hacking attacks, and therefore the risk of a data breach is severely increased [1,2]. Moreover, data centralization increases the risk of reidentification of deidentified data because of the increased data size [3].

To reduce the computational cost, Google proposed a method known as federated learning (FL), which uses the computational cores in mobile devices [4-6]. In FL, training is performed at the individual client level, and then the local weights of each client are sent to the server. The server collects the updated local weights and calculates the new global weights. Subsequently, the client downloads the global weights from the server and continues the training process. Since its first use in mobile apps [7-9], many researchers have been studying and improving FL in various fields [10-14]. In particular, studies on heterogeneity of data [4,15], robust optimization [16-20], and security methods such as differential privacy and secure multiparty computation have also been conducted with an FL approach [12,21,22]. Research on FL has also been conducted in the medical field [10,13,19]. In particular, studies have been conducted using electronic medical records and brain tumor data [23-25]. However, the application of FL to real medical data has not been sufficiently studied.

FL can be used to resolve privacy issues and mitigate the risk of a data breach in clinical information, since transfer and centralization of data are not required. Privacy protection is particularly beneficial for medical data analysis, since medical data represent some of the most sensitive types of personal data. To protect patients’ privacy, deidentification methods have typically been applied [26-28]. However, data centralization is required for both deidentifying data and evaluating the risk of reidentification. If the data are centralized, the risk of a data breach is increased. Moreover, when deidentifying the dataset, the direct or indirect identifiers in the medical data must be determined. This is challenging because of the lack of clear guidelines. The Health Insurance Portability and Accountability Act in the United States provides clear deidentification guidance; it defines 18 types of protected health information to be removed [29]. However, many researchers and social activists claim that this guidance should be revised to enhance privacy protection [30]. In contrast, FL does not require the centralization of raw data. As a result, even the FL developers cannot access the raw data. Therefore, FL can solve privacy or deidentification issues that occur when using clinical data.

### Objectives

The aim of this study was to assess the performance of FL on three benchmark datasets: the Modified National Institute of Standards and Technology (MNIST) dataset, Medical Information Mart for Intensive Care-III (MIMIC-III) dataset, and PhysioNet Electrocardiogram (ECG) dataset. We also verified FL in environments that simulate real-world data distributions by modifying the MNIST, MIMIC-III, and ECG datasets.

## Methods

### FL Code and Server

FL is supported by several open-source projects, including TensorFlow Federated in TensorFlow 2.0 [31], PySyft [32,33], and Federated AI Technology Enabler [34,35]. However, there are limitations in using these libraries. First, most of these libraries only support a single server and not a network environment. Therefore, there is no control process for data communication. Second, as a prototype, the necessary features were not fully implemented to handle a complex dataset. For future research using real clinical data from hospitals, we implemented our own client-server version of FL using Python. The implemented server code is available on the FL_Server repository [36] and the client code is available on the FL_Client repository [37]. The MNIST dataset analyzed during the current study is available in the Keras package in the TensorFlow framework. Additionally, the original code used to generate and preprocess the MIMIC-III experiment used in this study referred to the mimic3-benchmarks repository [38]. The original MIMIC-III dataset analyzed during this study is available on the PhysioNet repository [39]. The ECG dataset analyzed during this study is available on the 2017 PhysioNet/CinC Challenge website [40]. The model and environment assessed in this study refer to Hannun et al [41].

The FL server was developed using the Django framework and Python in Amazon Web Services (AWS). The server provides several application programming interfaces (APIs) for communication with a client, as shown in Table 1, and performs federated averaging (FedAVG) [4], which calculates the weighted averages. FedAVG is a widely used optimization algorithm that calculates the average value when the local weights collected from the client reach a specific level. The implemented code was deployed and managed in AWS Beanstalk, which was continuously monitored during the training process.

**Table 1:** Application programming interface calls provided by the server.

Method	URL	Parameter	Description	Return
GET	/round	N/A^a^	Request current round	Number
GET	/weight	N/A	Request global weight	List
PUT	/weight	List	Update local weight	N/A

^a^N/A: Not applicable.

### Client

The client consists of three components. The first is the local learning component, which builds a suitable model for the dataset during the learning phase. The second is the communication component, which updates local weights according to the results of local training (the first component) on the server and downloads the global weights from the server. The third is the performance measure component, in which the performance of each client is measured using the downloaded global weights. The implemented code was deployed on an AWS EC2 instance. We used the specifications of g4dn.xlarge with the NVIDIA T4 Tensor core GPU for the Amazon instance.

### Communications

Client–server communication for FL was implemented based on the process described by McMahan et al [42]. However, the implemented code exhibits some differences. The communication assumes that all clients (hospitals) are always powered (as is the case for a typical computer but not for a mobile device) and that their online status is maintained by a wired network connection. In addition, rather than selecting clients via an eligibility criterion from multiple client pools (thousands or millions), the code was implemented to manage a predefined fixed number of clients. In other words, all clients could participate in each round.

A schematic diagram of the FL client–server communication is shown in Multimedia Appendix 1. In brief, the client decides whether to participate in the current round through the API. If it has already participated (sending local weights to the server), it waits to participate in the next round. The server waits for the client’s weight updates and ensures that no clients are eventually dropped. All communications are performed through the API provided by the server. The monitoring system is used to continuously observe system abnormalities.

### Datasets

#### MNIST

The MNIST dataset, which consists of digit handwriting images, contained 70,000 samples (including 60,000 for training and 10,000 for testing). The basic model was a simple artificial neural network with an input layer, one hidden layer with 128 units with a rectified linear unit activation function, and an output layer. The hyperparameters for training were set as follows: batch size 32, maximum 1000 epochs, and early stopping. Stochastic gradient descent was used as an optimizer [43].

For FL, we used 10 individual clients to best mimic a real environment. We modified the datasets and hyperparameters of the learning algorithms. The datasets were modified considering differences in the distribution of medical data between hospitals. Hyperparameters were adjusted for training in each client. The proposed approach was evaluated on the MNIST dataset in four different experiments.

We first evaluated the basic performance of the FL. Ten clients randomly selected 600 images from the basic dataset. We continued the process for up to 500 rounds and observed the results. For the imbalanced FL experiment, each client used different sizes of randomly selected data, ranging from 1 to 600, for training (ie, one client used 36 data points and another client used 537 data points). However, other environments such as hyperparameters and the number of rounds were the same as set in the basic FL experiment. In addition, the MNIST dataset was split into single-digit groups, ranging from 0 to 9. Each of the 10 numbers was assigned to 10 different clients. Consequently, each client had a single digit instead of 10. This modified MNIST simulated an extremely skewed data distribution. Each client randomly selected 600 images from a dataset with a single digit for training. The simple artificial neural network used in the basic model was also used in these experiments. The hyperparameters were set as follows: 5 epochs and a batch size of 10. We continued the process for up to 3000 rounds and observed the results. For evaluation, a model was created with the latest updated global weights using 10,000 test samples. Finally, we conducted an extension of the modified MNIST FL that represents a skewed distribution. Each client was trained on data with an imbalanced and skewed distribution. Hence, each client was trained only on a single digit using a randomly selected sample.

#### MIMIC-III

The MIMIC-III dataset is a clinical dataset related to human health information, including demographics, vital signs, laboratory tests, and medications from intensive care units. MIMIC-III data were preprocessed using a state-of-the-art (SOTA) benchmark [44]. In this case, FL experiments with three individual clients were performed to predict in-hospital mortality, which is a classification problem that predicts death within the first 48 hours of an intensive care unit stay. After preprocessing the MIMIC-III dataset using the method described by Harutyunyan et al [44], the dataset contained 21,139 samples (including 17,903 for training and 3236 for testing). The basic model was a standard long short-term memory (LSTM) with reference to the benchmark [44]. The LSTM was chosen with 16 hidden units, depth 2, dropout 0.3, time step 1.0, batch size 8, and an adaptive moment estimation (ADAM) optimizer.

For FL, randomly chosen samples from the original dataset were divided into 3 datasets without duplication and assigned to each client. This simulates having data from three different institutions. The same basic LSTM was used, and hyperparameters were set as follows: 2 epochs and a batch size of 4. We continued the process for up to 30 rounds and observed the results.

For the basic FL experiment, each client was trained on a subset of data that were split into three parts with the same data size without duplication. For the imbalanced FL experiment, all data were split into 50%, 30%, and 20% without duplication, and one subset was assigned to each client.

#### ECG

The 2017 PhysioNet/CinC Challenge ECG dataset was used in this study [40]. This target problem is a multiclassification problem that classifies four signals: atrial fibrillation, normal sinus rhythm, alternative rhythm, and noisy using a single short ECG signal. The total data size is 8528 single-lead ECG data points. The dataset was divided into 90% training data (7676) and 10% test data (852). For traditional learning, a convolution neural network with 34 layers based on Hannun et al [41] was applied to the ECG dataset. The hyperparameters were chosen with a batch size of 32 and an ADAM optimizer.

For FL, randomly chosen samples from the original dataset were divided into 3 datasets without duplication and assigned to 3 clients. The same model was used, and hyperparameters were set as follows: 3 epochs and a batch size of 16. We continued the process for up to 30 rounds and observed the results.

For the basic FL experiment, each client was trained on a subset of data that were split into three parts with the same data size without duplication. For the imbalanced FL experiment, all data were split into 50%, 30%, and 20% without duplication, and a subset was assigned to each client.

### Evaluation

During training, we monitored the FL accuracy to evaluate performance. If the accuracy did not improve during the round, we completed the FL. Finally, we chose the best model and conducted bootstrapping to determine if there were significant differences between the experiments.

In all experiments, the area under the receiver operating characteristic curve (AUROC) score and F1-score were used as performance metrics. In addition, we evaluated the confusion matrix, precision, recall, or area under the precision recall curve (AUPRC) for comparison with the performance of the SOTA method. We calculated the 95% CIs and resampled the test set *K* times (for MNIST and ECG, *K* was 100, whereas for MIMIC-III, *K* was 10,000).

## Results

### MINST

The proposed approach was evaluated on the MNIST dataset for five different cases (as described in the Methods). Table 2 presents the values of the AUROC and F1-score for each case, and Multimedia Appendix 2 presents the confusion matrix for each case.

**Table 2:** Comparison of the experimental results for the five different MNIST cases described in the Methods.^a^

Experiments	AUROC^b^ (95% CI)	F1-score (95% CI)	Precision (95% CI)	Recall (95% CI)
CML^c^	0.999 (0.999-0.999)	0.981 (0.978-0.983)	0.981 (0.972-0.989)	0.981 (0.971-0.989)
Basic FL^d^	0.997 (0.996-0.998)	0.946 (0.941-0.950)	0.945 (0.929-0.959)	0.945 (0.930-0.959)
Imbalanced FL	0.995 (0.994-0.995)	0.921 (0.917-0.927)	0.920 (0.904-0.937)	0.920 (0.903-0.937)
Skewed FL	0.992 (0.991-0.993)	0.905 (0.899-0.911)	0.905 (0.885-0.922)	0.904 (0.885-0.920)
Imbalanced and skewed FL	0.990 (0.989-0.991)	0.891 (0.884-0.896)	0.890 (0.869-0.909)	0.889 (0.868-0.908)

^a^All experiments used the same model and hyperparameters. All results are presented with a 95% CI by resampling the validation task 100 times.

^b^AUROC: area under the receiver operating characteristic curve.

^c^CML: centralized traditional machine-learning method.

^d^FL: federated learning.

Centralized machine learning (CML) is a baseline training method that was used as a control group. CML achieved an AUROC of 0.999 and an F1-score of 0.981. For basic FL, the AUROC and F1-score were 0.997 and 0.946, respectively. The initial performance of the basic FL was fairly high, with an accuracy of approximately 0.800, which continually improved (Multimedia Appendix 3A).

Imbalanced FL was designed to reflect a realistic clinical data distribution. As described in the Methods section, each client had a different training data size. Interestingly, the performance of imbalanced FL was significantly superior, with an AUROC and F1-score of 0.995 and 0.921, respectively. The initial performance was rather poor, as expected. However, after several rounds of processing, the performance rapidly improved to reach an accuracy of 0.900, after which the performance improvement was slow (Multimedia Appendix 3B).

Skewed FL assumed an extreme case. Each client had only one digit from 0 to 9, thereby simulating a situation in which each hospital has a unique subpopulation of patients without overlaps. The final AUROC and F1-score were 0.992 and 0.905, respectively. As expected, the initial performance was poor; however, it rapidly improved after the initial rounds (Multimedia Appendix 3C).

The most extreme case was designed by combining an imbalanced and a skewed dataset. In this experiment, the AUROC and F1-score were 0.990 and 0.891, respectively. Similar to the skewed FL, the initial performance was very poor, but it rapidly improved after the initial rounds (Multimedia Appendix 3D).

Additionally, the precision and recall results for each digit class classification in each experiment are presented in Multimedia Appendices 4-8.

### MIMIC-III

The proposed approach was evaluated on the MIMIC-III dataset in two different cases to compare the performance with a reported benchmark. FL experiments were performed on three individual clients. Apart from the AUROC and F1-score, we also refer to the AUPRC, which is reported in the benchmark [44]. The results are presented in Table 3 and in Multimedia Appendices 9 and 10.

SOTA performance was achieved by executing the codes provided in Harutyunyan

[38]. FL achieved an AUROC, F1-score, and AUPROC comparable with those of the SOTA method. The imbalanced FL experiment, as an extension of the basic MIMIC-III FL, also achieved AUROC, F1-score, and AUPRC comparable with those of SOTA (Table 3).

**Table 3:** Comparison results of MIMIC-III.^a^

Experiments	AUROC^b^ (95% CI)	F1-score (95% CI)	AUPRC^c^ (95% CI)	Precision (95% CI)	Recall (95% CI)
SOTA^d^	0.857 (0.837-0.875)	0.944 (0.938-0.950)	0.505 (0.451-0.558)	0.973 (0.967-0.979)	0.773 (0.907-0.927)
Basic FL^e^	0.850 (0.830-0.869)	0.944 (0.938-0.950)	0.483 (0.427-0.537)	0.975 (0.969-0.980)	0.797 (0.906-0.926)
Imbalanced FL	0.850 (0.829-0.869)	0.943 (0.937-0.949)	0.481 (0.426-0.535)	0.981 (0.976-0.986)	0.714 (0.897-0.918)

^a^All results are presented with a 95% CI by resampling 10,000 times.

^b^AUROC: area under the receiver operating characteristic curve.

^c^AUPRC: area under the precision-recall curve.

^d^SOTA: state of the art.

^e^FL: federated learning.

### ECG

The proposed approach was evaluated on the ECG database using two different methods to compare the performance with a reported benchmark [41]. The results are presented in Table 4 and Multimedia Appendices 11-14.

Benchmark results were achieved using the code available on github [45]. The AUROC and F1-score of both basic and imbalanced FL were comparable with those of the benchmark (Table 4).

**Table 4:** Comparison results for the electrocardiogram dataset.^a^

Experiments	AUROC^b^ (95% CI)	F1-score (95% CI)	Precision (95% CI)	Recall (95% CI)
Benchmark	0.954 (0.930-0.978)	0.814 (0.655-0.910)	0.820 (0.672-0.943)	0.814 (0.640-0.936)
Basic FL^c^	0.938 (0.860-0.978)	0.807 (0.651-0.931)	0.823 (0.645-0.942)	0.795 (0.660-0.925)
Imbalanced FL	0.943 (0.883-0.977)	0.807 (0.635-0.902)	0.830 (0.650-0.935)	0.788 (0.626-0.905)

^a^All results are presented with a 95% CI by resampling 100 times.

^b^AUROC: area under the receiver operating characteristic curve.

^c^FL: federated learning.

## Discussion

### Principal Findings

When comparing the performances of CML and FL in basic MNIST experiments, both the AUROC and F1-score were high. Unexpectedly, when using an imbalanced dataset, FL delivered good performance with only small differences (AUROC and F1-score of 0.003 and 0.035, respectively). When using a skewed dataset, FL also yielded remarkable results with respect to both the AUROC and F1-score. When comparing the confusion matrices for experiments with four datasets (ie, normal, imbalanced, skewed, and a combination of two distributions), FL showed some deterioration in performance for visually similar numbers (eg, 3 vs 5; 4 vs 9). Even in the basic MNIST classification, the performance was relatively poor in these cases. However, this problem was not related to the small sizes of the training datasets. When we monitored the size of the training datasets for each client, the dataset for class 5 was not small. Moreover, depending on the experiment, the datasets for class 1 or 7 could be small, but superior classification performance was nevertheless achieved. This trend was maintained in the experiments with basic FL and imbalanced FL using the MIMIC-III dataset.

The FL experiments using MIMIC-III also exhibited good and competitive performance compared to a benchmark that has been trained on CML. The experimental results of in-hospital mortality using the MIMIC-III dataset, which is a well-known dataset with real clinical data, also showed good performance. This experiment was performed by splitting the randomly selected MIMIC-III data into three parts (ie, from the perspective of each institution, learning one-third of the total data). However, the performances of FL and CML were almost the same, with only a 0.005 difference in AUROC detected compared with the SOTA performance reported by Harutyunyan et al [44]. Before the experiments, we expected that the performance of FL would be slightly inferior to that of CML because FL uses a distributed dataset instead of a centralized dataset. Nevertheless, no significant difference was found in well-known evaluation indicators such as accuracy, sensitivity, precision, and F1-score (except for AUROC). Experimental results with an imbalanced dataset were very similar to those of basic FL. Therefore, an individual client may only use a small amount of data for training in FL, and the results will be similar to those achieved when all available data are used for training.

The FL experiments using ECG data also exhibited good and competitive performance compared to CML. This experiment was performed by splitting the randomly selected ECG data into three parts (ie, from the perspective of each institution, learning on one-third of the total data) with each using different data distributions.

However, the performance of FL and CML was not significantly different. Experimental results with an imbalanced dataset were very similar to those for basic FL. As shown in Multimedia Appendices 12-14, the noisy case was shown to have relatively low performance in precision and recall. This is because the data size for training was only 3% of the total size. However, the other classes performed well, such as atrial fibrillation, normal sinus rhythm, and alternative rhythm.

The performance of FL was verified using three datasets with changed data distributions: imbalanced (with disproportionally represented classes) and skewed (the distribution of the target variable was different) to imitate real-world medical data. As a result, FL was comparable to CML. During the initial rounds, only a relatively small amount of data was used on each client instead of an ensemble; therefore, the performance of FL was significantly inferior to that of CML. However, in the subsequent rounds, the performance of FL (with respect to AUROC and F1-score) became similar to that of CML. Typically, medical centers have datasets with very different distributions, and our results demonstrate that FL is suitable for real-world medical datasets without requiring data centralization.

One reason for the comparable performance of FL might be that the weight updates and the process of FedAVG could have a similar effect in mini-batches [46-48] and ensembles [49]. In FL, each client trains on a relatively small dataset and then transfers the local weights to the server. The server then collects the local weights and updates the global weights that reflect all of the data through FedAVG. Subsequently, the round is repeated to improve the global weights. Hence, individual clients are an element of a mini-batch, and FedAVG is similar to ensemble processing. When implementing FL, we used the widely known FedAVG aggregation method [5], but this does not guarantee the best choice. To solve this problem, many researchers have studied aggregation methods that can work well with abnormal distributions, robust aggregation, and efficient communication such as FedProx [16], FSVRG [17], CO-OP [18], LoAdaBoost FedAVG [19], and RFA [20]. Hyperparameter selection also requires further research.

In addition, many researchers have studied methods to reduce communication costs. First, it has been suggested to reduce the communication round through methods such as client selection, peer-to-peer, and local update [11-13]. Second, a method such as sparsification, subsampling, or quantization has been suggested to reduce the communication message size [12,13]. Third, the asynchronous update method in traditional parallel computation methods can be applied.

FL can be used to build medical artificial intelligence apps by protecting patient privacy. Although the data themselves are not exposed or gathered in the central repository in FL, these data can nevertheless be guessed during the aggregation process in the network [12]. Therefore, other privacy preservation methods such as differential privacy, secure multiparty computation, and homomorphic encryption [11,12,21,22] might be necessary to protect privacy from diverse up-to-date privacy attack methods.

In future studies, we plan to use the proposed FL methods in real clinical datasets rather than benchmark datasets. First, we will try to improve the FL framework based on the results from this study. We will then compare the performance of a breast cancer recurrence prediction model using data from two different medical centers in Korea.

### Conclusions

Our experiments demonstrated the potential of FL in terms of performance and data protection, which is important for dealing with sensitive medical data. Specifically, in FL, only weights are transferred, and the participants are unaware of each other’s local datasets. This can prevent personal information leaks. In addition, the proposed approach can be used to supplement existing approaches and to avoid problems that may occur during the deidentification process. The future direction of research is to use FL for actual medical data through collaborations with multiple institutions. Tasks such as expanding the client–server version of FL and improving communication will be expected to be important for the application of FL in real-world medical data with multiple institutions.

## Appendix

Multimedia Appendix 1 Client–server communication logic.

Multimedia Appendix 2 Confusion matrices for the MNIST experiments.

Multimedia Appendix 3 Accuracy changes for each round of MNIST federated learning (FL) experiments.

Multimedia Appendix 4 Each digit class classification results of precision and recall in a centralized machine learning (CML) experiment using the MNIST dataset.

Multimedia Appendix 5 Each digit class classification result of precision and recall in the basic federated learning (FL) experiment using the MNIST dataset.

Multimedia Appendix 6 Each digit class classification result of precision and recall in the imbalanced federated learning (FL) experiment using the MNIST dataset.

Multimedia Appendix 7 Each digit class classification result of precision and recall in the skewed federated learning (FL) experiment using the MNIST dataset.

Multimedia Appendix 8 Each digit class classification result of precision and recall in an imbalanced and skewed federated learning (FL) experiment using the MNIST dataset.

Multimedia Appendix 9 Area under the receiver operating characteristic curve, which is the result of the in-hospital mortality prediction for each experiment.

Multimedia Appendix 10 Confusion matrix of federated learning (FL) to predict in-hospital mortality using MIMIC-III. (A) Basic FL. (B) Imbalanced FL.

Multimedia Appendix 11 Confusion matrices for the ECG experiments.

Multimedia Appendix 12 Each class classification result of precision and recall in a centralized machine learning (CML) experiment using the ECG dataset.

Multimedia Appendix 13 Each class classification result of precision and recall in the basic federated learning (FL) experiment using the ECG dataset.

Multimedia Appendix 14 Each class classification result of precision and recall in the imbalanced federated learning (FL) experiment using the ECG dataset.

## Abbreviations

ADAM: adaptive moment estimation
API: application programming interface
AUPRC: area under the precision-recall curve
AUROC: area under the receiver operating characteristic curve
AWS: Amazon Web Services
CML: centralized machine learning
ECG: electrocardiogram
FedAVG: federated averaging
FL: federated learning
LSTM: long short-term memory
MIMIC-III: Medical Information Mart for Intensive Care III
MNIST: Modified National Institute of Standards and Technology
SOTA: state of the art
