# Supplementary figures and images for: Federated Learning on Clinical Benchmark Data: Performance Assessment
Source: J Med Internet Res. 2020 Oct 26;22(10):e20891. doi: 10.2196/20891 (PMC7652692; doi:10.2196/20891)

## Multimedia Appendix 1. Server-Client communication logic

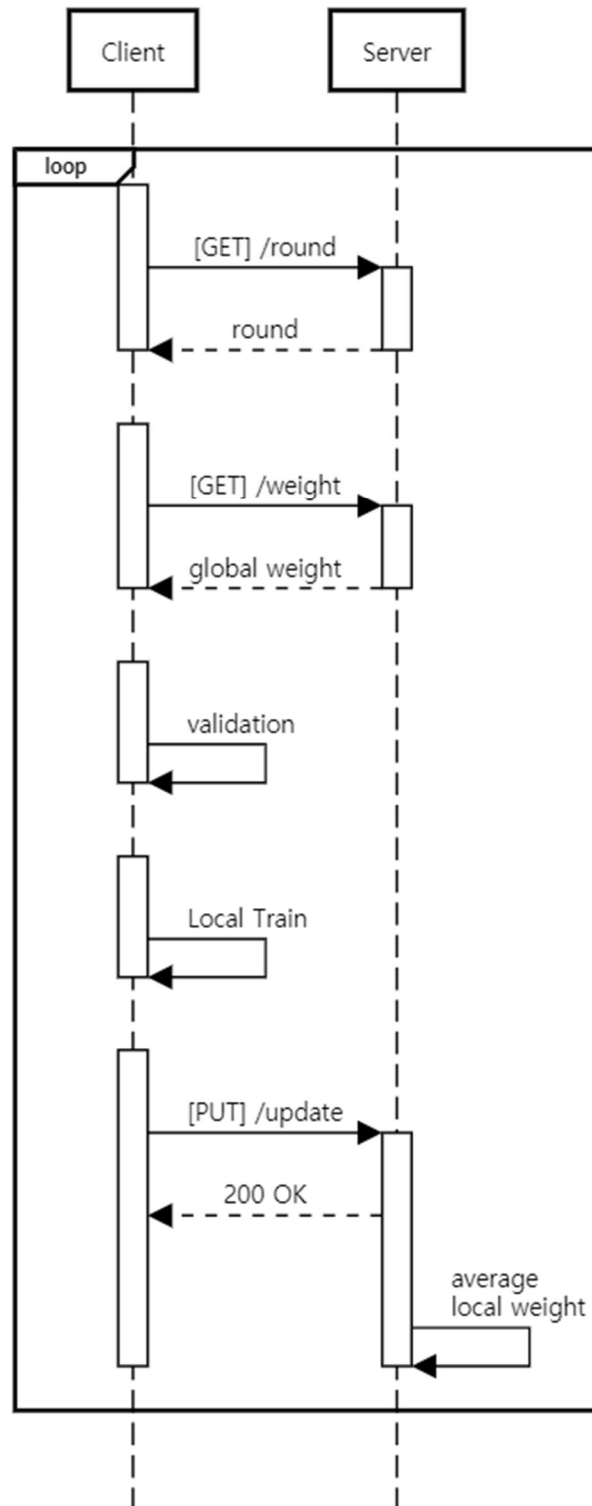

Supplement: Multimedia Appendix 1 [file jmir_v22i10e20891_app1.pdf]
