# Supplementary material for: Federated Learning on Clinical Benchmark Data: Performance Assessment
Source: J Med Internet Res. 2020 Oct 26;22(10):e20891. doi: 10.2196/20891 (PMC7652692; doi:10.2196/20891)
Supplement: Multimedia Appendix 2 [file jmir_v22i10e20891_app2.pdf]

Multimedia Appendix 2. Confusion matrices for the MNIST experiments. (A) Centralized machine learning (CML). (B) Basic FL. (C) Imbalanced FL. (D) Skewed FL. (E) Imbalanced and skewed FL.

969	0	1	0	0	2	2	2	2	2
0	1,121	3	1	0	1	3	2	4	0
4	2	1,012	2	2	0	2	4	4	0
0	0	2	992	0	4	0	5	4	3
3	0	3	0	952	0	3	2	2	12
3	0	0	7	1	868	6	1	4	2
5	3	2	1	5	3	939	0	0	0
0	2	8	4	0	0	0	1,007	2	5
6	0	3	5	6	5	4	4	938	3
3	2	0	6	13	2	1	7	6	969

A

967	0	1	1	0	3	4	1	1	2
0	1,122	2	1	0	1	5	1	3	0
5	2	998	6	3	1	4	8	5	0
0	0	6	983	0	5	1	7	5	3
2	0	3	1	950	0	5	2	2	17
5	1	0	13	1	853	9	1	6	3
5	3	1	1	7	5	936	0	0	0
1	9	10	4	3	1	0	991	0	9
3	2	5	7	5	7	3	4	938	0
4	5	1	7	17	2	1	5	3	964

B

960	0	1	1	0	7	8	1	2	0
0	1,112	5	3	0	2	3	1	9	0
10	3	935	26	11	3	8	13	20	3
5	1	15	917	1	28	1	12	22	8
1	5	7	1	903	0	15	3	4	43
9	4	6	33	10	778	12	6	18	16
24	1	14	0	13	14	889	0	3	0
1	17	25	6	7	0	0	946	2	24
6	8	15	21	16	16	12	6	867	7
5	9	1	8	34	13	1	26	6	906

C

967	0	0	1	0	2	8	1	1	0
0	1,107	4	2	0	1	4	2	15	0
14	12	901	10	9	2	19	18	42	5
6	2	30	875	0	33	4	24	26	10
4	1	12	0	888	0	11	3	12	51
17	8	8	41	11	724	24	13	37	9
11	3	6	0	7	6	921	1	3	0
3	10	31	2	3	0	0	949	3	27
12	12	13	17	9	26	18	15	841	10
10	8	2	7	39	6	1	47	10	879

D

957	0	2	3	0	3	14	1	0	0
0	1,078	7	4	0	2	4	1	39	0
19	10	899	8	7	2	34	9	33	11
8	2	29	883	1	23	4	18	35	7
5	0	2	0	863	1	39	5	8	59
30	9	2	65	7	682	30	17	41	9
10	3	4	1	9	9	917	1	4	0
2	18	30	5	7	0	0	931	4	31
20	6	7	24	9	28	22	10	835	13
15	8	0	10	49	7	2	46	12	860

E
