# Supplementary material for: Federated Learning on Clinical Benchmark Data: Performance Assessment
Source: J Med Internet Res. 2020 Oct 26;22(10):e20891. doi: 10.2196/20891 (PMC7652692; doi:10.2196/20891)
Supplement: Multimedia Appendix 3 [file jmir_v22i10e20891_app3.pdf]

Multimedia Appendix 3. Accuracy changes for each round of MNIST federated learning (FL) experiments. (A). Basic FL. (B) Imbalanced FL. (C) Skewed FL. (D) Imbalanced and skewed FL.

A

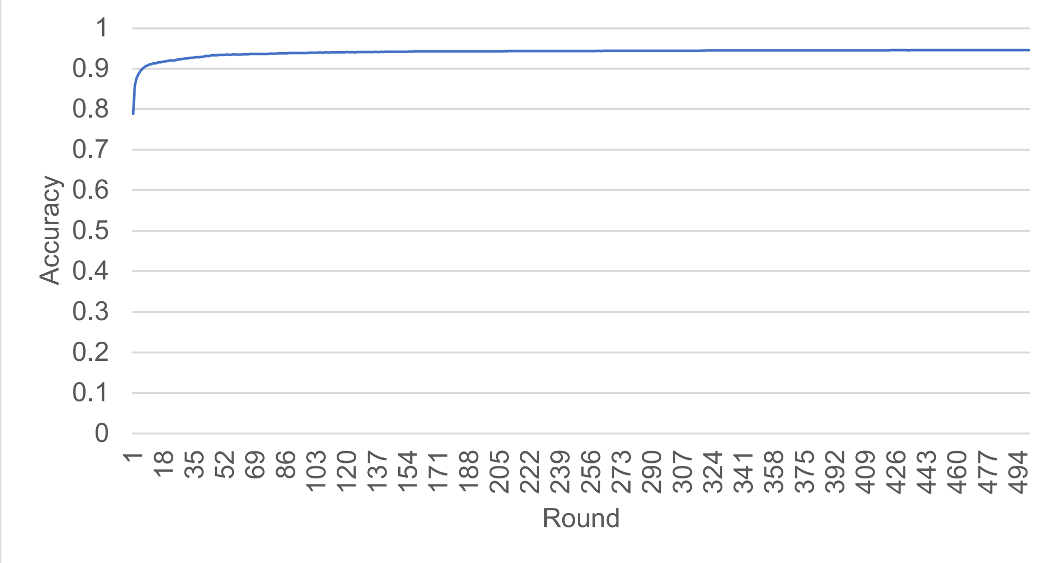

B

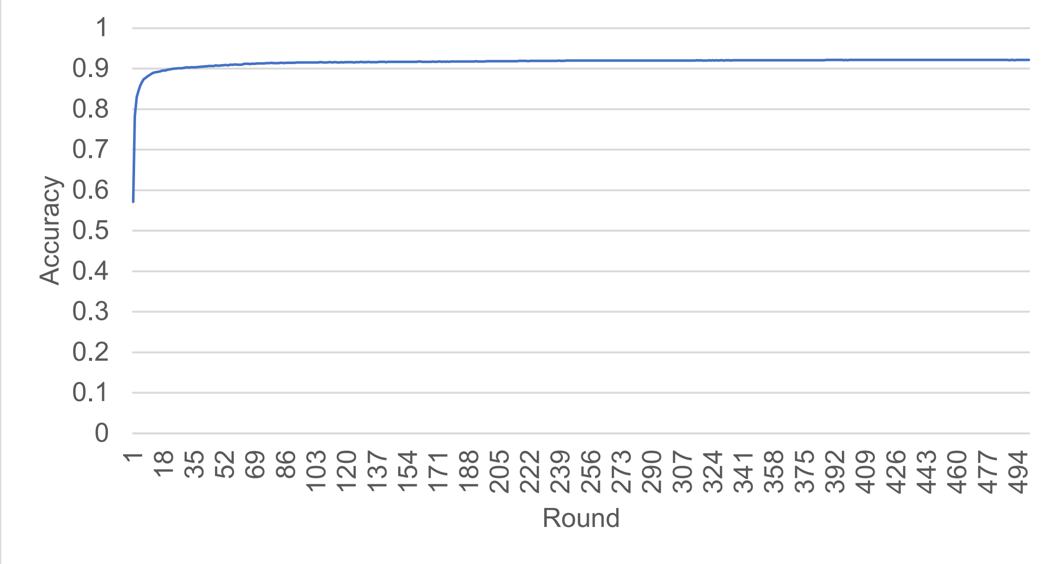

C

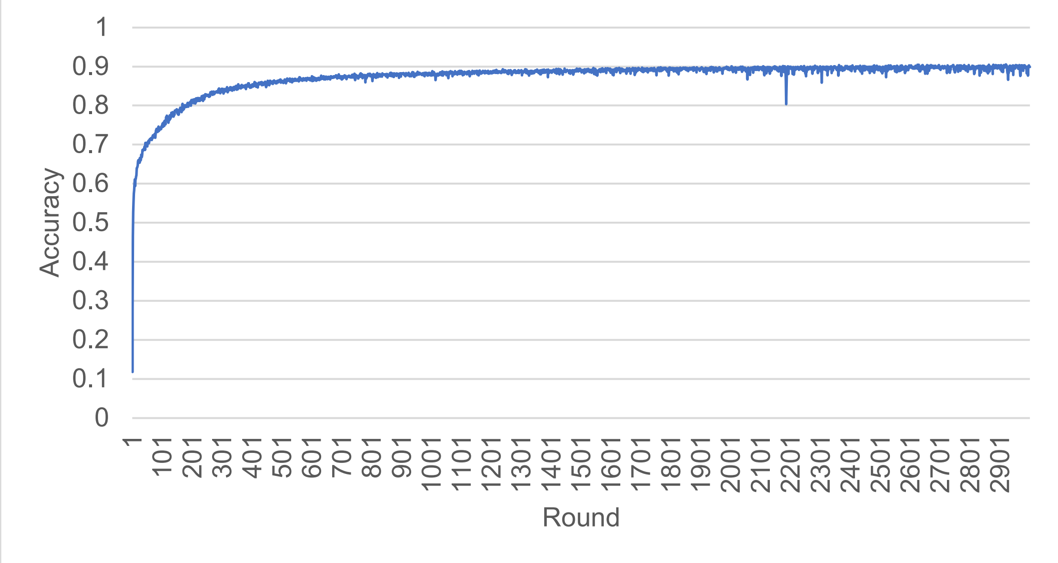

D	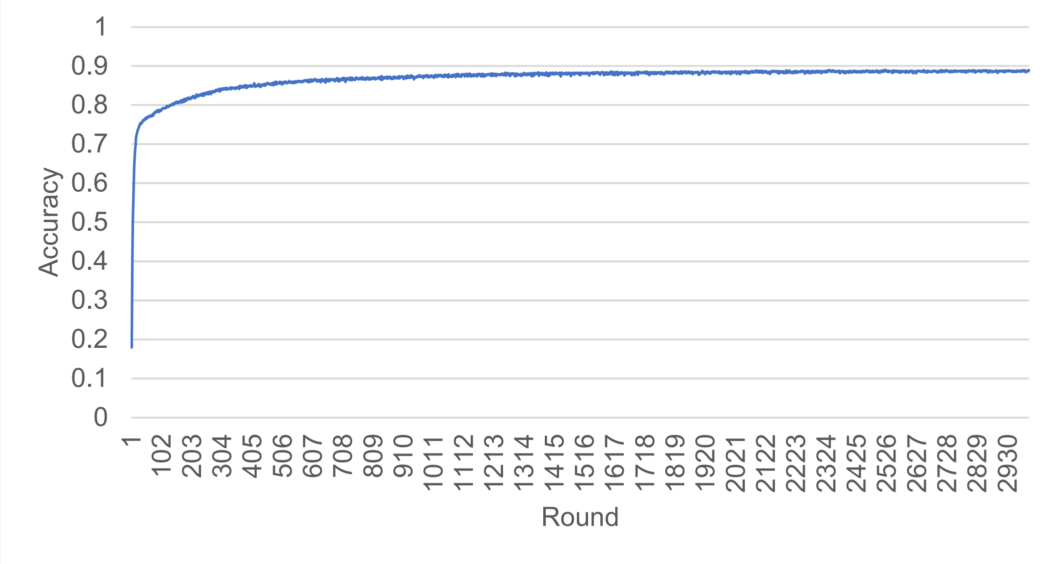 Accuracy<br>1<br>0.9<br>0.8<br>0.7<br>0.6<br>0.5<br>0.4<br>0.3<br>0.2<br>0.1<br>0<br>1<br>102<br>203<br>304<br>405<br>506<br>607<br>708<br>809<br>910<br>1011<br>1112<br>1213<br>1314<br>1415<br>1516<br>1617<br>1718<br>1819<br>1920<br>2021<br>2122<br>2223<br>2324<br>2425<br>2526<br>2627<br>2728<br>2829<br>2930<br>Round
