# Supplementary material for: Federated Learning on Clinical Benchmark Data: Performance Assessment
Source: J Med Internet Res. 2020 Oct 26;22(10):e20891. doi: 10.2196/20891 (PMC7652692; doi:10.2196/20891)
Supplement: Multimedia Appendix 4 [file jmir_v22i10e20891_app4.pdf]

Multimedia Appendix 4. Each digit class classification results of precision and recall in a centralized machine learning (CML) experiment using the MNIST dataset. All results are presented with a 95% confidence interval by resampling the validation task 100 times.

CML	Precision	Recall
0	0.986 (0.955, 1.000)	0.988 (0.961, 1.000)
1	0.989 (0.966, 1.000)	0.993 (0.973, 1.000)
2	0.976 (0.943, 1.000)	0.985 (0.956, 1.000)
3	0.977 (0.943, 1.000)	0.985 (0.954, 1.000)
4	0.983 (0.953, 1.000)	0.981 (0.948, 1.000)
5	0.985 (0.955, 1.000)	0.977 (0.942, 1.000)
6	0.981 (0.948, 1.000)	0.984 (0.955, 1.000)
7	0.982 (0.949, 1.000)	0.970 (0.932, 1.000)
8	0.983 (0.953, 1.000)	0.970 (0.932, 1.000)
9	0.972 (0.935, 1.000)	0.979 (0.946, 1.000)
