# Supplementary material for: Federated Learning on Clinical Benchmark Data: Performance Assessment
Source: J Med Internet Res. 2020 Oct 26;22(10):e20891. doi: 10.2196/20891 (PMC7652692; doi:10.2196/20891)
Supplement: Multimedia Appendix 5 [file jmir_v22i10e20891_app5.pdf]

Multimedia Appendix 5. Each digit class classification result of precision and recall in the Basic FL experiment using the MNIST dataset. All results are presented with a 95% confidence interval by resampling the validation task 100 times.

Basic FL	Precision	Recall
0	0.955 (0.910, 0.990)	0.981 (0.952, 1.000)
1	0.970 (0.938, 1.000)	0.989 (0.966, 1.000)
2	0.944 (0.898, 0.981)	0.939 (0.883, 0.980)
3	0.931 (0.875, 0.976)	0.942 (0.887, 0.982)
4	0.942 (0.892, 0.980)	0.947 (0.899, 0.989)
5	0.946 (0.890, 0.989)	0.913 (0.850, 0.966)
6	0.938 (0.885, 0.980)	0.962 (0.917, 1.000)
7	0.954 (0.905, 0.990)	0.930 (0.874, 0.975)
8	0.941 (0.888, 0.981)	0.926 (0.866, 0.976)
9	0.938 (0.884, 0.978)	0.925 (0.870, 0.972)
