# Supplementary material for: Federated Learning on Clinical Benchmark Data: Performance Assessment
Source: J Med Internet Res. 2020 Oct 26;22(10):e20891. doi: 10.2196/20891 (PMC7652692; doi:10.2196/20891)
Supplement: Multimedia Appendix 6 [file jmir_v22i10e20891_app6.pdf]

Multimedia Appendix 6. Each digit class classification result of precision and recall in the Imbalanced FL experiment using the MNIST dataset. All results are presented with a 95% confidence interval by resampling the validation task 100 times.

Imbalanced FL	Precision	Recall
0	0.942 (0.895, 0.981)	0.981 (0.951, 1.000)
1	0.960 (0.918, 0.992)	0.980 (0.952, 1.000)
2	0.914 (0.856, 0.966)	0.907 (0.848, 0.956)
3	0.902 (0.840, 0.955)	0.908 (0.846, 0.959)
4	0.906 (0.847, 0.958)	0.919 (0.859, 0.968)
5	0.904 (0.835, 0.961)	0.873 (0.792, 0.940)
6	0.936 (0.883, 0.979)	0.928 (0.872, 0.974)
7	0.934 (0.882, 0.978)	0.921 (0.864, 0.969)
8	0.911 (0.848, 0.965)	0.888 (0.824, 0.947)
9	0.899 (0.833, 0.952)	0.898 (0.835, 0.952)
