# Supplementary material for: Federated Learning on Clinical Benchmark Data: Performance Assessment
Source: J Med Internet Res. 2020 Oct 26;22(10):e20891. doi: 10.2196/20891 (PMC7652692; doi:10.2196/20891)
Supplement: Multimedia Appendix 7 [file jmir_v22i10e20891_app7.pdf]

Multimedia Appendix 7. Each digit class classification result of precision and recall in the Skewed FL experiment using the MNIST dataset. All results are presented with a 95% confidence interval by resampling the validation task 100 times.

Skewed FL	Precision	Recall
0	0.927 (0.872, 0.971)	0.988 (0.961, 1.000)
1	0.951 (0.906, 0.990)	0.977 (0.944, 1.000)
2	0.895 (0.833, 0.949)	0.873 (0.810, 0.932)
3	0.915 (0.856, 0.966)	0.867 (0.790, 0.931)
4	0.918 (0.863, 0.968)	0.904 (0.838, 0.958)
5	0.905 (0.837, 0.962)	0.810 (0.728, 0.882)
6	0.912 (0.851, 0.960)	0.962 (0.918, 1.000)
7	0.885 (0.821, 0.944)	0.923 (0.867, 0.971)
8	0.850 (0.778, 0.919)	0.861 (0.784, 0.926)
9	0.887 (0.822, 0.944)	0.871 (0.805, 0.929)
