# Supplementary material for: Federated Learning on Clinical Benchmark Data: Performance Assessment
Source: J Med Internet Res. 2020 Oct 26;22(10):e20891. doi: 10.2196/20891 (PMC7652692; doi:10.2196/20891)
Supplement: Multimedia Appendix 8 [file jmir_v22i10e20891_app8.pdf]

Multimedia Appendix 8. Each digit class classification result of precision and recall in an imbalanced and skewed FL experiment using the MNIST dataset. All results are presented with a 95% confidence interval by resampling the validation task 100 times.

Imbalanced and skewed FL	Precision	Recall
0	0.899 (0.833, 0.955)	0.978 (0.947, 1.000)
1	0.951 (0.906, 0.985)	0.950 (0.909, 0.984)
2	0.916 (0.856, 0.969)	0.873 (0.804, 0.932)
3	0.880 (0.812, 0.939)	0.876 (0.804, 0.939)
4	0.906 (0.847, 0.960)	0.878 (0.805, 0.937)
5	0.901 (0.826, 0.969)	0.764 (0.674, 0.845)
6	0.860 (0.792, 0.922)	0.957 (0.911, 0.991)
7	0.897 (0.830, 0.954)	0.906 (0.845, 0.959)
8	0.825 (0.755, 0.899)	0.855 (0.775, 0.920)
9	0.869 (0.795, 0.932)	0.851 (0.782, 0.913)
