# Supplementary material for: Federated Learning on Clinical Benchmark Data: Performance Assessment
Source: J Med Internet Res. 2020 Oct 26;22(10):e20891. doi: 10.2196/20891 (PMC7652692; doi:10.2196/20891)

Multimedia Appendix 9. Area under the receiver operating characteristic curve, which is the result of the in-hospital mortality prediction for each experiment, that is, basic and imbalanced FL, using the MIMIC-III dataset.

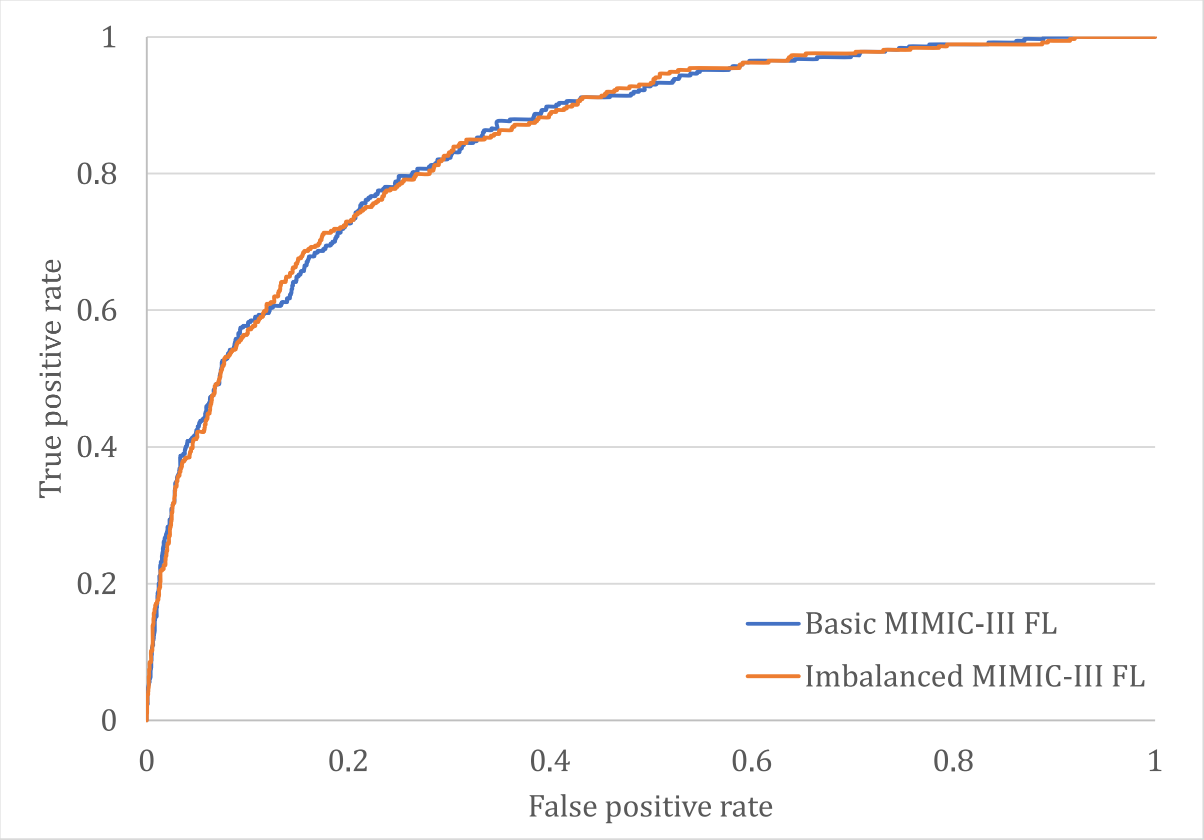

Supplement: Multimedia Appendix 9 [file jmir_v22i10e20891_app9.pdf]
