# Supplementary material for: Federated Learning on Clinical Benchmark Data: Performance Assessment
Source: J Med Internet Res. 2020 Oct 26;22(10):e20891. doi: 10.2196/20891 (PMC7652692; doi:10.2196/20891)

Multimedia Appendix 10. Confusion matrix of federated learning (FL) to predict in-hospital mortality using MIMIC-III. (A) Basic FL. (B) Imbalanced FL.

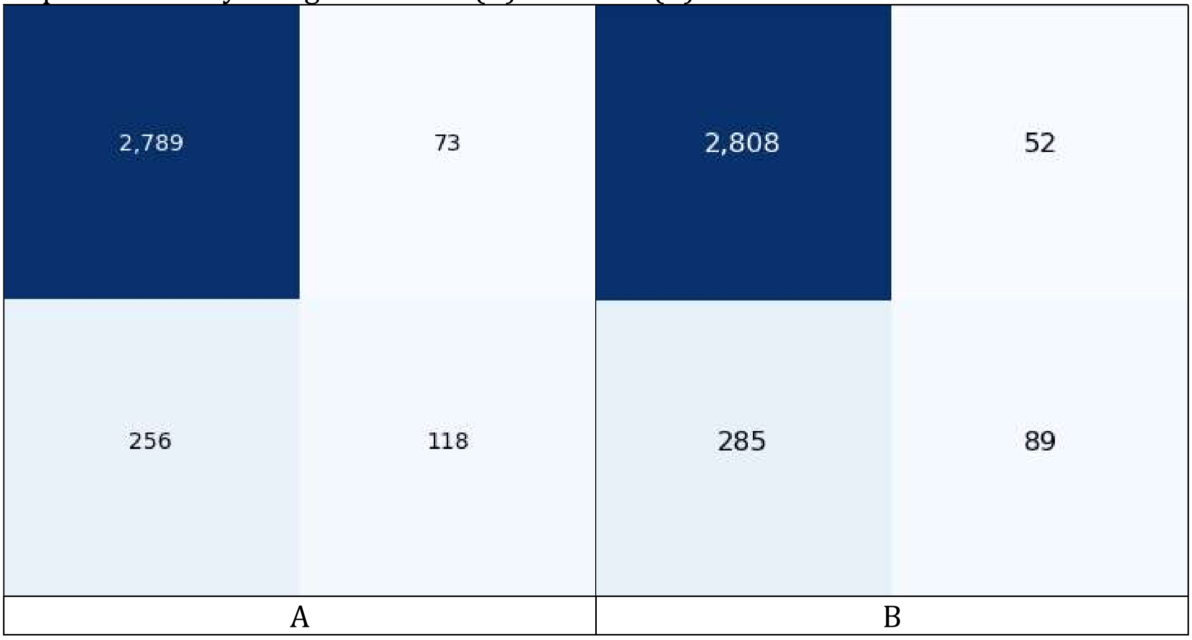

Supplement: Multimedia Appendix 10 [file jmir_v22i10e20891_app10.pdf]
