# Supplementary material for: Federated Learning on Clinical Benchmark Data: Performance Assessment
Source: J Med Internet Res. 2020 Oct 26;22(10):e20891. doi: 10.2196/20891 (PMC7652692; doi:10.2196/20891)
Supplement: Multimedia Appendix 11 [file jmir_v22i10e20891_app11.pdf]

Multimedia Appendix 11. Confusion matrices for the ECG experiments. (A) Centralized machine learning (CML). (B) Basic FL. (C) Imbalanced FL. Each class name is as follows: A - atrial fibrillation, N - normal sinus rhythm, O - alternative rhythm, and ~ - noisy.

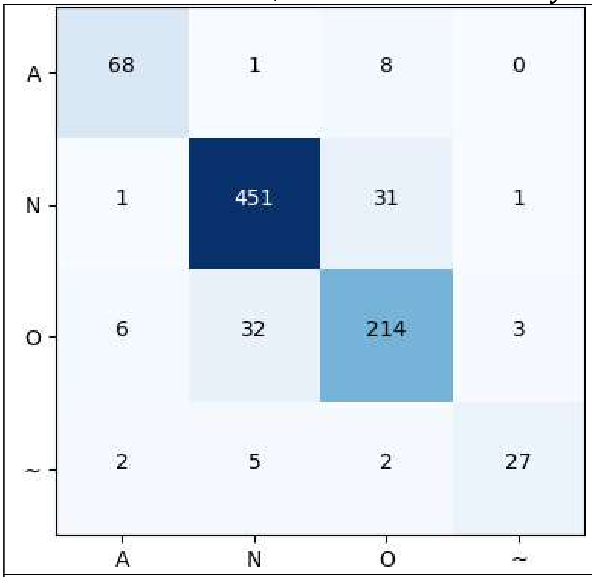

A

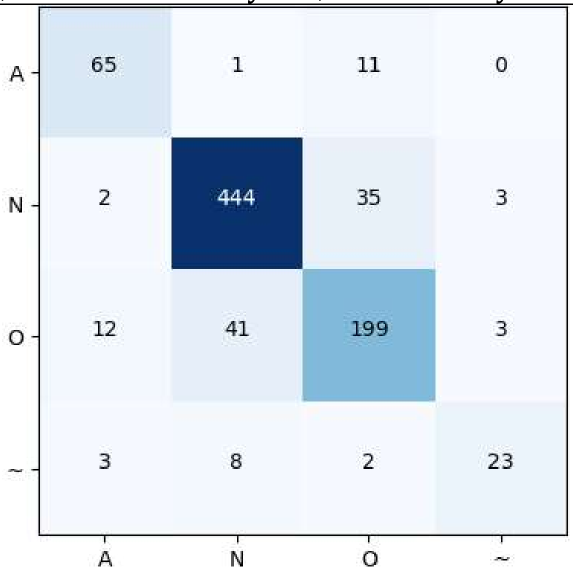

B

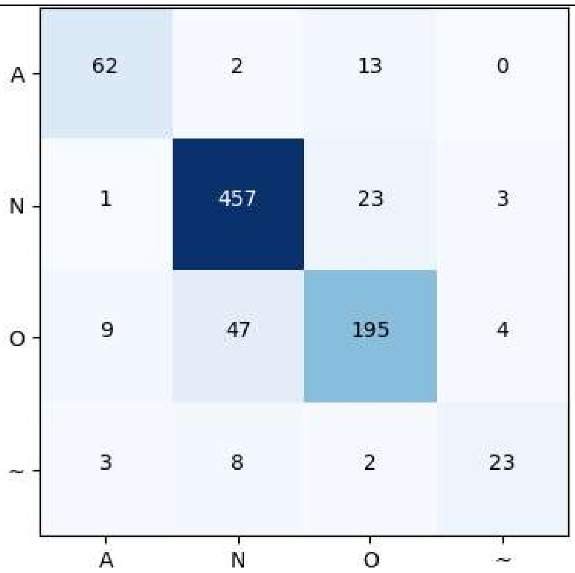

C
