# Supplementary material for: Federated Learning on Clinical Benchmark Data: Performance Assessment
Source: J Med Internet Res. 2020 Oct 26;22(10):e20891. doi: 10.2196/20891 (PMC7652692; doi:10.2196/20891)
Supplement: Multimedia Appendix 12 [file jmir_v22i10e20891_app12.pdf]

Multimedia Appendix 12. Each class classification result of precision and recall in a centralized machine learning (CML) experiment using the ECG dataset. All results are presented with a 95% confidence interval by resampling the validation task 100 times.

CML	Precision	Recall
atrial fibrillation	0.914 (0.667, 1.000)	0.795 (0.545, 1.000)
normal sinus rhythm	0.900 (0.825, 0.966)	0.931 (0.885, 0.983)
alternative rhythm	0.820 (0.667, 0.925)	0.779 (0.630, 0.914)
noisy	0.685 (0.250, 1.000)	0.781 (0.333, 1.000)
