# Supplementary material for: Federated Learning on Clinical Benchmark Data: Performance Assessment
Source: J Med Internet Res. 2020 Oct 26;22(10):e20891. doi: 10.2196/20891 (PMC7652692; doi:10.2196/20891)
Supplement: Multimedia Appendix 13 [file jmir_v22i10e20891_app13.pdf]

Multimedia Appendix 13. Each class classification result of precision and recall in the Basic FL experiment using the ECG dataset. All results are presented with a 95% confidence interval by resampling the validation task 100 times.

Basic FL	Precision	Recall
atrial fibrillation	0.806 (0.556, 1.000)	0.876 (0.625, 1.000)
normal sinus rhythm	0.904 (0.815, 0.965)	0.919 (0.846, 0.984)
alternative rhythm	0.808 (0.640, 0.938)	0.787 (0.656, 0.931)
noisy	0.821 (0.333, 1.000)	0.637 (0.250, 1.000)
