# Supplementary material for: Federated Learning on Clinical Benchmark Data: Performance Assessment
Source: J Med Internet Res. 2020 Oct 26;22(10):e20891. doi: 10.2196/20891 (PMC7652692; doi:10.2196/20891)
Supplement: Multimedia Appendix 14 [file jmir_v22i10e20891_app14.pdf]

Multimedia Appendix 14. Each class classification result of precision and recall in the Imbalanced FL experiment using the ECG dataset. All results are presented with a 95% confidence interval by resampling the validation task 100 times.

Imbalanced FL	Precision	Recall
atrial fibrillation	0.841 (0.600, 1.000)	0.828 (0.626, 0.905)
normal sinus rhythm	0.890 (0.783, 0.954)	0.943 (0.889, 1.000)
alternative rhythm	0.833 (0.684, 0.960)	0.767 (0.633, 0.914)
noisy	0.782 (0.250, 1.000)	0.613 (0.200, 1.000)
